# Proprioceptive drift in the rubber hand illusion is intensified following 1 Hz TMS of the left EBA

**DOI:** 10.3389/fnhum.2014.00390

**Published:** 2014-06-04

**Authors:** Andrew Wold, Jakub Limanowski, Henrik Walter, Felix Blankenburg

**Affiliations:** ^1^Berlin School of Mind and Brain, Humboldt UniversityBerlin, Germany; ^2^Neurocomputation and Neuroimaging Unit, Freie Universität BerlinBerlin, Germany; ^3^Division of Mind and Brain Research, Charité University of MedicineBerlin, Germany

**Keywords:** rubber hand illusion, transcranial magnetic stimulation, extrastriate body area, body representation, proprioceptive drift

## Abstract

The rubber hand illusion (RHI) is a paradigm used to induce an illusory feeling of owning a dummy hand through congruent multisensory stimulation. Thus, it can grant insights into how our brain represents our body as our own. Recent research has demonstrated an involvement of the extrastriate body area (EBA), an area of the brain that is typically implicated in the perception of non-face body parts, in illusory body ownership. In this experiment, we sought causal evidence for the involvement of the EBA in the RHI. Sixteen participants took part in a sham controlled, 1 Hz repetitive transcranial magnetic stimulation (rTMS) experiment. Participants received (RHI condition) or asynchronous (control) stroking and were asked to report the perceived location of their real hand, as well as the intensity and the temporal onset of experienced ownership of the dummy hand. Following rTMS of the left EBA, participants misjudged their real hand’s location significantly more toward the dummy hand during the RHI than after sham stimulation. This difference in “proprioceptive drift” provides the first causal evidence that the EBA is involved in the RHI and subsequently in body representation and further supports the view that the EBA is necessary for multimodal integration.

## INTRODUCTION

The rubber hand illusion (RHI) is a well-established paradigm to manipulate the sense of body ownership in healthy individuals (Botvinick and Cohen, 1998). When one’s own occluded hand and an anatomically congruent dummy hand are stroked synchronously, this leads to a feeling of ownership over the dummy hand that is generally interpreted as a momentary incorporation of the seen dummy hand into the participant’s body representation (Ehrsson et al., 2004; Tsakiris and Haggard, 2005). The RHI has been explained as a result of multisensory information integration in a hierarchically organized cortical network that ultimately constructs and maintains one’s body representation (Hohwy, 2007; Makin et al., 2007; Tsakiris, 2010; Blanke, 2012; Limanowski and Blankenburg, 2013; Apps and Tsakiris, 2014). Thus, it is assumed that visual, proprioceptive, and somatosensory input feed into higher-order multimodal integration areas (Tsakiris, 2010; Blanke, 2012). Previous research, however, has indicated that visual areas work merely on low level processing such as representing visual form (Makin et al., 2007), whereas recent research has emphasized some of these visual areas as playing a more sophisticated role in body representation (Ionta et al., 2011; Gentile et al., 2013; Limanowski et al., 2014), particularly focusing on the so-named extrastriate body area (EBA; Downing et al., 2001).

The EBA is an occipito-temporal visual region that has gained considerable attention in recent literature because of its selective, strong response to non-face body parts (Downing et al., 2001; Peelen and Downing, 2007; Downing and Peelen, 2011) and contribution to explicit representations of identity (Urgesi et al., 2007), body configurations (Pitcher et al., 2009), and goal-directed actions (Wiggett and Downing, 2011). Along these lines, a recent fMRI study by Limanowski et al. (2014) demonstrated an involvement of the EBA in illusory body ownership. The involvement of the EBA in the RHI provides support for previous speculations about a role of this region in the representation of one’s body (Costantini et al., 2011; Apps and Tsakiris, 2014).

In this experiment, we sought causal evidence for the EBA’s role in body ownership by applying repetitive transcranial magnetic stimulation (rTMS) over the left EBA during the RHI (following Limanowski et al., 2014, who found ownership-related activity changes in the left EBA, contralateral to the arm subjected to the RHI). TMS to the EBA has causally proven the EBA’s pivotal role in non-face body part perception (Urgesi et al., 2004). Previous studies applying TMS over the left inferior parietal lobe (IPL; Kammers et al., 2009) and right temporo-parietal junction (TPJ; Tsakiris et al., 2008) have successfully modulated behavioral measurements of the RHI, but until now no one has explored this combination with regard to the EBA.

We hypothesized that, if the EBA is indeed involved in the processing of one’s body representation, interfering with its neural activity during illusions of body ownership should result in significant changes on the behavioral measures of the RHI, namely verbal reports, and the so-named “proprioceptive drift,” the relative displacement of the perceived location of one’s own hand toward the location of the rubber hand after the RHI, compared with a pre-stimulation baseline (e.g., Tsakiris and Haggard, 2005).

## MATERIALS AND METHODS

### PARTICIPANTS

Nineteen participants (11 female, median age: 25, range: 21–42) took part in this experiment. Three participants (two female) did not experience the illusion, and were therefore excluded after the first session. That 16 of 19 participants did experience is in accordance with classic RHI literature estimating the illusion to function in approximately 80% of the population (Botvinick and Cohen, 1998). All participants gave written informed consent to participate in the study, which had been approved by the local University Hospital Ethics Committee (Charité - Universitätsmedizin Berlin) and was within limits of safety guidelines (Rossi et al., 2009).

### DESIGN AND PROCEDURE

**FIGURE 1:**
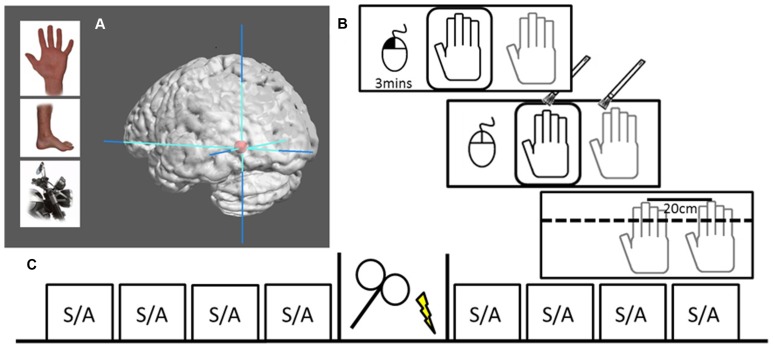
**Localization and design. (A)** Examples of body parts and motorcycle parts used to functionally localize the EBA. Graphic brain rendering represent mean peak activation of left EBA (mean MNI-coordinates: -45, -76, -11) surrounded by max stereotactic error in red (10 mm), image rendered using Mango Imaging software [Research Imaging Institute, University of Texas Health Science Center at San Antonio (UTHSCSA)]. **(B)** RHI set-up where the dummy hand can be seen through viewing window (black) while actual hand is hidden under table (gray). The left hand controls a computer mouse, beginning the 3 min session with a left button press. Hand stroking is applied for 3 min, during which participants voluntarily make a left mouse button press to signal that they experience the illusion. At the end of stimulation the viewing window is covered and participants make three perceptual judgments followed by a subjective experience rating. **(C)** The design of one session, including four pre-TMS blocks of either (S)ynchronous or (A)synchronous stoking, 20 min of either rTMS or sham stimulation, and four post-TMS stroking blocks.

In a two session, single-blind, sham-controlled, counterbalanced crossover design, participants received either 20 min of real or sham 1 Hz rTMS (1200 pulses) over their left EBA, which was functionally defined by a standard EBA localizer in a separate fMRI session (see **Figure 1**). The participants were seated in front of a meter long table with a window that gave them full view of a realistic right dummy hand. The table was set at an angle of 15°, had an additional opaque layer to cover the entire surface. The participant’s right hand was positioned at 20 cm distance from the dummy hand. Participants were instructed to keep their right hand still throughout the experiment and were observed by the experimenter, who only proceeded if this was actually the case. The experiment began with nine proprioceptive judgments (as a calibration to obtain a subject-specific baseline against which the following judgments were compared), then four randomly assigned stroking blocks (two synchronous, two asynchronous) comprised the pre- and post-stimulation sessions. Each stroking session lasted 3 min beginning by participant’s performing a button click with a computer mouse under their left hand. During stroking, participants were instructed to click again when they experienced the RHI onset. Hand stroking was delivered with paintbrushes by the experimenter, at an approximate frequency of 1 Hz, and included vertical stroking (from knuckle to finger-tip) of fingers and horizontal stroking (from left most knuckle to right most). Asynchronous stroking was displaced both temporally and spatially. Following the stroking block, participants were asked for three drift measurements and to rate the intensity of the illusion. Participants were asked to maintain focus on the dummy hand during stroking and to look away from the measuring tape between proprioceptive trials; so that they would not focus on any particular spot on the set-up. Total time for an entire block was approximately 3.5 min. After the four pre-stimulation blocks, participants moved into a different chair in the same room to receive real or sham TMS, and then repeated the same procedure of calibration followed by another four 3.5-min blocks. Motor threshold (MT) was only assessed during the first session.

### rTMS TARGET SELECTION AND STEREOTACTIC NAVIGATION

All participants had their left EBA’s localized via a 3 Tesla MRI scanner (Magnetom Tim Trio, Siemens, Erlangen, Germany) with a 32-channel head coil. Using a standard EBA localizer task, two 6-min sessions of eight randomly assigned blocks of body parts or motorcycle parts were presented to participants. In each task session, 175 functional volumes were acquired with a T2^*^-weighted EPI-sequence, each consisting of 37 oblique-axial slices (voxel size = 3 mm × 3 mm × 3 mm, 64 × 64 matrix, FOV = 192 mm, TR = 2000 ms, TE = 30 ms, flip angle = 70°). After the functional runs, a high-resolution T1-weighted structural image was acquired for each participant (3D MPRAGE, voxel size = 1 mm × 1 mm × 1 mm, FOV = 256 mm × 256 mm, 176 slices, TR = 1900 ms, TE = 2.52 ms, flip angle = 9°). Six subjects already had their EBA localized in an fMRI experiment we conducted recently (Limanowski et al., 2014). The functional as well as anatomical data for these subjects were taken from this previous study. Data were preprocessed and analyzed using SPM 8 (Wellcome Department of Cognitive Neurology, London, UK) and BrainVoyager (Brain Innovation B.V., Netherlands).

After motion correction, participant’s functional images were coregistered to their respective structural images and smoothed with a 5-mm full width at half maximum Gaussian kernel, but were not normalized. For each subject, the two sessions were included in one general linear model and the contrast body parts minus motorcycle parts was computed to localize EBA (i.e., activity specific to vision of body parts). The localization was individually derived for each participant. Six of our participants came from a previous study (Limanowski et al., 2014) but utilized non-normalized data for navigation. Statistical parametric maps of the body parts versus motorcycle parts were thresholded at a significance threshold of *p* < 0.01 to *p* < 0.001 uncorrected. This anatomical and functional data were imported from SPM into BrainVoyager QX, aligned in the AC–PC space, and marked with a target file corresponding to the individual left EBA (via peak activation of the EBA localizer). Using this target file and head mesh reconstructions of the participants brain, we then coregistered TMS coil and head position using a Zebris CMS20S tracking device (Zebris Medical GmbH, Isny, Germany), allowing us to navigate coil position relative to target, in real-time. After the localization session the data were converted into Talairach space using the normalization procedure in BrainVoyager. The Talairach coordinates where then transformed to MNI-coordinates using the TAL2MNI Matlab code (tal2icbm_spm; retrieved from www.brainmap.org/icbm2tal/tal2icbm_spm.m). The mean MNI-coordinates (±SEM) for the EBA of the 16 participants corresponded to *x* = -45 ± 0.95, *y* = -76 ± 1.9, *z* = -11 ± 1.7, which also align to previously published locations of the EBA (Downing et al., 2001).

### TRANSCRANIAL MAGNETIC STIMULATION

Transcranial magnetic stimulation was applied using Magstim Rapid^2^ device (Magstim, Whitland, UK). In order to establish the appropriate intensity of stimulation, resting MT of the left hemisphere was determined according to the standard MT procedure (mean ± SEM = 56.2 ± 1.3% of maximum stimulator output; Schutter and van Honk, 2006). Two stimulation intensities were used: a high- and a low-intensity rTMS (80 and 40% of the MT) for a total of 1200 pulses at a frequency of 1 Hz. The figure-of-eight coil was turned tangentially to the scalp and the handle aligned along the rostrocaudal plane. The low-intensity rTMS application (assumed to be neurally ineffective) served as the sham condition for non-specific effects of rTMS, such as the “click” sound and the scalp sensation inevitably associated with rTMS delivery (Auksztulewicz et al., 2011). Full stimulation was lowered from 100% of MT to reduce the current spread into neighboring cortical regions (Fitzgerald et al., 2006).

### MEASUREMENTS

First, proprioceptive drift was assessed by having participants report a number on a measuring tape that best corresponded to the perceived location of the index finger of their hidden right hand. Three measurements were always taken while the dummy hand was hidden from view by an additional opaque layer, on top of which the measuring tape was placed. The experimenter had a list of randomized displacements (ranging 1–20 cm) at which to hold a standard measuring tape and instructed the participant to verbalize the felt position of the right index finger to the nearest centimeter. Before the RHI was induced, the set-up was calibrated with nine perceptual judgments before the pre- and post-stimulation block. Mean calibrations (±SEM) for all participants did not differ significantly between the pre- and the post-stimulation sessions (-0.63 ± 0.66 cm, and -0.63 ± 0.82 cm, relative to actual finger position). Second, participants were asked to rate the intensity of the ownership illusion after each stroking block. The question was “How strongly do you feel that this hand could belong to your own body?” on a 7-point Likert scale from -3 (strongly disagree) to 0 (unsure of what I felt) to +3 (strongly agree). Third, for each stimulation block, participants were instructed to make a mouse click with their left hand as soon as they experienced the ownership illusion, if they experienced it at all. This time point was taken to represent the temporal onset of illusory ownership (see Ehrsson et al., 2004).

### CALCULATIONS AND DATA ANALYSIS

All behavioral data were analyzed with SPSS (version 12.2) software. Proprioceptive drift was calculated per block as the mean of the three measurements minus the individual’s calibration value (relative to the edge of set-up), making pre-stroking subjectively felt finger location the proprioceptive reference. The rating was reported as a value from -3 to +3. Onset was calculated as the total duration minus the onset (i.e., 180 s - onset), so that higher values represent faster responses to RHI. For each measurement of each pre- and post-stimulation session, the two synchronous and asynchronous blocks were separately averaged.

Data were analyzed using repeated measures MANOVA and ANOVA (all measurements passed a Shapiro–Wilk test for normality). *Post hoc* paired *t*-tests were calculated to compare real TMS to sham TMS, significance was assessed using a Bonferroni corrected alpha level of α = 0.05 and two-tailed distributions.

## RESULTS

**FIGURE 2:**
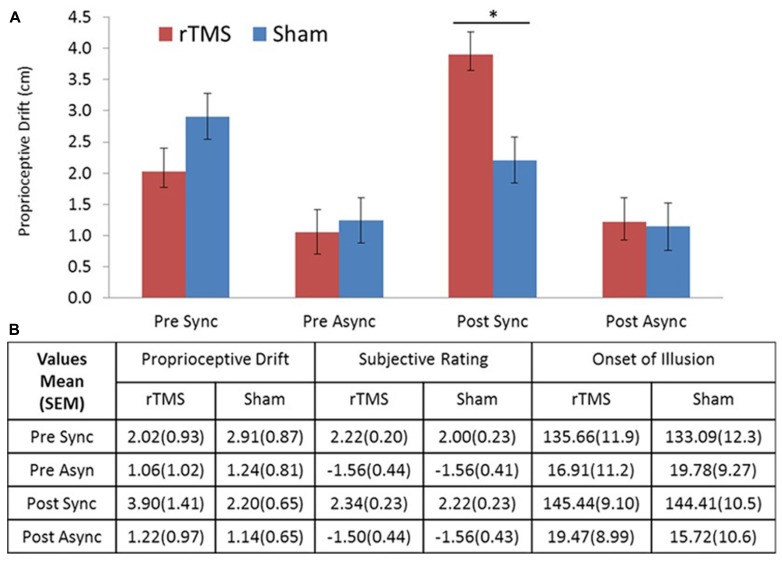
**(A)** Drift results. Proprioceptive drift in centimeters showed a significant difference between rTMS and sham stimulation for post-TMS sync stroking. **(B)** Table of descriptive statistics. Means and standard errors of the mean (SEM) for proprioceptive drift, ownership rating, and illusion onset (s). Sync, synchronous; Async, asynchronous stroking. All error bars represent SEM, star represents significance at α = 0.05 (Bonferroni corrected).

All participants completed the entire experiment and had no adverse side effect associated with TMS (for descriptive statistics, see **Figure 2B**). The repeated measures MANOVA including three measurements (proprioceptive drift, rating, and illusion onset), two levels of stimulation (rTMS/sham), two levels of session (pre/post stimulation), and two levels of stroking condition (synchronous/asynchronous) showed a significant main effect for measurement [*F*(2,30) = 108.9, *p* < 0.001] and stroking condition [*F*(2,30) = 107.8, *p* < 0.001]. In addition, this analysis revealed a significant four-way interaction between stimulation test × session × stroking condition [*F*(2,14) = 8.475, *p* = 0.004]. To make the analysis more comprehensible, we computed three-way repeated measure ANOVAs for each of the individual measures. These analyses revealed that the three-way interaction (stimulation × session × stroking condition) in proprioceptive drift was significant [*F*(1,15) = 15.29, *p* < 0.001], whereas the corresponding three-way interactions in ownership rating or illusion onset were not [*F*(1,15) = 0.256, *p* = 0.620 and *F*(1,15) = 0.409, *p* = 0.532, respectively]. A closer inspection of the significant interaction in proprioceptive drift (see **Figure 2**) elucidates the effect of increased drift in the synchronous stroking condition following rTMS. Based on our assumption that rTMS stimulation would produce an effect compared to sham stimulation during synchronous stroking but not asynchronous stroking, we compared proprioceptive drift in the post-stimulation session using *post hoc* paired *t*-tests, which revealed a significantly (*p* < 0.025) stronger effect of rTMS versus sham stimulation on proprioceptive drift during synchronous stroking [*t*(15) = 2.578, *p* = 0.021], but not during asynchronous stroking [*t*(15) = 0.161, *p* = 0.876].

Since it has been shown that men and women express different EBA functional lateralization effects in body perception (Aleong and Paus, 2010), we ran the repeated measure MANOVA with gender as a between subjects factor. This analysis revealed no significant effect of gender [*F*(1,15) = 0.001, *p* = 0.976], neither did further repeated measures ANOVAs with gender as between subjects variable for the individuals RHI measurements [proprioceptive drift: [*F*(1,15) = 0.103, *p* = 0.753; ownership rating: *F*(1,15) = 0.022, *p* = 0.883; illusion onset: *F*(1,15) = 0.001, *p* = 0.995].

## DISCUSSION

Repetitive TMS over the left EBA, compared with sham stimulation, resulted in an increased proprioceptive drift in the RHI (synchronous stroking of the rubber hand and real hand) versus the control condition (asynchronous stroking). Ownership rating and illusion onset, like drift, could be differentiated by stroking condition (synchronous > asynchronous), but unlike proprioceptive drift were not differently affected by rTMS versus sham stimulation. Together, our results suggest a causal involvement of the left EBA in the processing of an own body representation, as discussed in the following.

The EBA has been shown to be causally involved in non-face body perception (Urgesi et al., 2004) and the processing of haptic and visual information (Costantini et al., 2011). In this way, the EBA may be part of a hierarchical network of brain areas – and due to its potentially multimodal processing capacities likely located at intermediate levels in this hierarchy – representing the own body in a probabilistic fashion (Hohwy, 2013; Limanowski and Blankenburg, 2013; Apps and Tsakiris, 2014). Moreover, Jackson et al. (2006) propose that the EBA is not only important for the visual processing of body parts but mapping that representation of another body onto one’s own body. Our results – an increased behavioral effect of illusory limb ownership – may be seen as support for this view. The causal modulation of EBA activity by rTMS may increase the drift toward the rubber hand, not by confusion of the location of one’s own hand, but by allowing an illusory body part to be incorporated into one’s body representation.

Proprioceptive drift is a multimodal measure combining the processing of visual, tactile, and proprioceptive information (Botvinick and Cohen, 1998; Tsakiris and Haggard, 2005). Tsakiris (2010) proposes that there is a pre-existing body model containing a reference description of the visual, anatomical and structural properties of the body, and that during the RHI certain multimodal brain regions (e.g., TPJ) act as comparators, matching the (dummy) viewed hand with the body model reference. Modulating this comparator process has an effect on proprioceptive drift, as is evident in rTMS experiments stimulating the rTPJ during the RHI (Tsakiris et al., 2008). In our study, rTMS over EBA also affected drift, but led to an increase (versus a decreased drift found by Tsakiris et al., 2008). This result suggests that the EBA might be also involved in this process of body model-comparison, potentially integrating visual body representations with somatosensory and proprioceptive information with regard to body parts (Costantini et al., 2011; Blanke, 2012; Apps and Tsakiris, 2014). It should be noted that we only found an effect of rTMS over left EBA on proprioceptive drift but not the verbal ownership ratings or onsets. This could be because of ceiling effects of the reported illusion onsets or the ownership rating scale not being sensitive enough to discriminate fine differences in subjective experience. However, as has been previously reported, mechanisms of RHI measurements are different and may not actually be associated, as is documented in the case of the dissociation between drift and ownership (Holmes et al., 2006; Rohde et al., 2011). Future work will have to investigate the exact role of EBA in the construction and maintenance of one’s body model – most importantly it will have to be clarified whether EBA acts as a multimodal integration region and/or as a region creating a body reference in terms of visual appearance and location.

As mentioned above our study is not the only one to combine RHI with TMS. However, this study differs from previous studies by stimulating the (left) EBA during RHI and by showing an increase in proprioceptive drift due to a TMS intervention. Kammers et al. (2009) stimulated the IPL showing that IPL affects the perceptual, rather than the sensorimotor, representation of the body. This was in accordance with neuroimaging studies showing the IPL to be involved in the RHI and hypothesized to play a role in the perception of size and location (Ehrsson et al., 2005). The rTMS results of Kammers et al. (2009) yielded a similar magnitude of effect but in the opposite direction (i.e., reduced drift). Tsakiris et al. (2008) stimulated the rTPJ, an area known for its role in perspective taking (Saxe and Wexler, 2005) and multisensory integration (Tsakiris and Haggard, 2005). Following online single pulse TMS, Tsakiris et al. (2008) also found reduced drift compared to sham stimulation. It should be noted that the RHI controls and calculations differ among these studies, but we suggest that the EBA is not acting on the RHI through perception of size and location, like the IPL. Moreover, the difference in TMS effect on the EBA and regions such as the TPJ could reflect a difference in the underlying cortical mechanism used to process a sense of bodily self. Arzy et al. (2006) distinguishes between the left EBA and right TPJ for neural mechanisms of embodiment (shortly defined as the sense of being localized within one’s physical body), showing that the EBA is more present for embodied processing while the TPJ is distinct to disembodied processing. This difference could be the basis for the TMS effect observed, which we would assume is a reduced ability to embody one’s own hand or to visually discriminate the rubber hand from one’s own (i.e., increasing drift due to improved acceptance of rubber hand) when stimulating the EBA and a reduced ability to actualize a disembodied perspective (in the case of the RHI: reduced ability to self-attribute a foreign body part) when stimulating the TPJ. It should be noted that research shows bilateral TPJ activation in out-of-body-experience (Blanke et al., 2005) and self-location tasks (i.e., first-person perspective; Ionta et al., 2011). When distinguishing the TPJ and EBA in terms of their role for embodiment processing, one should therefore pay attention to hemispheric lateralization; our findings hence have to be interpreted with some caution as we only stimulated left EBA and cannot therefore reveal the relative role of EBA functional specialization and lateralization. Although time restraints limited the amount of rating questions in our studies, future work may benefit from including additional ratings pertaining to other aspects of the RHI besides ownership (e.g., increased visual similarity between real and fake hand; discussed as “perceptual assimilation” in Longo et al., 2008). Our null finding with regard to gender suggests that our data were not affected by the known gender difference in EBA functional specialization (Aleong and Paus, 2010), a larger sample would be needed to address the role of gender specific effects of the EBAs. Future studies focusing on these issues may help to uncover the differential EBA effects noted in our RHI experiment.

In sum, our study shows an increase in proprioceptive drift due to an rTMS intervention over left EBA; this increased mislocalization of one’s real hand position provides evidence for a causal involvement of the EBA in changes in one’s own body representation.

## AUTHOR CONTRIBUTIONS

Andrew Wold, Jakub Limanowski, and Felix Blankenburg developed the concept, Andrew Wold, Henrik Walter, Felix Blankenburg, and Jakub Limanowski contributed to the design, Andrew Wold and Jakub Limanowski conducted the experiment, Andrew Wold did the analysis, and all co-authors contributed to the manuscript.

## Conflict of Interest Statement

The authors declare that the research was conducted in the absence of any commercial or financial relationships that could be construed as a potential conflict of interest.
